# Comparative evaluation of intensified short course regimen and standard regimen for adults TB meningitis: a protocol for an open label, multi-center, parallel arms, randomized controlled superiority trial (INSHORT trial)

**DOI:** 10.1186/s13063-024-08133-6

**Published:** 2024-05-02

**Authors:** Leeberk Raja Inbaraj, Abi Manesh, C. Ponnuraja, Adhin Bhaskar, Vignes Anand Srinivasalu, Bella Devaleenal Daniel

**Affiliations:** 1 Department of Clinical Research, ICMR- National Institute for Research in Tuberculosis, Chethpet, Chennai, 600031 India; 2 Department of Infectious Diseases, Christian Medical College, Vellore, India; 3 Department of Statistics, ICMR- National Institute for Research in Tuberculosis, Chethpet, Chennai, 600031 India

**Keywords:** Randomized controlled trial, Tuberculous meningitis, High-dose rifampicin, Moxifloxacin, Intensified regimen, Aspirin

## Abstract

**Background:**

Despite several incremental improvements in the management of tuberculous meningitis (TBM), the mortality rates remain high. In spite of national and international guidelines, variation in the choice, dose, and duration of drugs exist between countries and clinicians. We propose to evaluate a shorter and more effective regimen containing agents with augmented intracerebral drug exposure and anti-inflammatory approaches to improve disability-free survival among patients with TBM. Our strategy incorporates the various developments in the field of TBM over the last two decades and only few trials have evaluated a composite of these strategies in the overall outcomes of TBM.

**Methods:**

An open label, parallel arms, randomized controlled superiority trial will be conducted among 372 participants across 6 sites in India. Eligible participants will be randomly allocated in 1:1:1 ratio into one of the three arms. The intervention arm consists of 2 months of high-dose rifampicin (25 mg/kg), moxifloxacin (400 mg), pyrazinamide, isoniazid, aspirin (150 mg), and steroids followed by rifampicin, isoniazid, and pyrazinamide for 4 months. The second intervention arm includes all the drugs as per the first arm except aspirin and the patients in the control arm will receive treatment according to the National TB Elimination Program guidelines. All participants will be followed up for 1 year after the treatment.

**Discussion:**

Current WHO regimens have agents with poor central nervous system drug exposure and is too long. It does not reflect the accumulating evidence in the field. We propose a comprehensive clinical trial incorporating the emerging evidence accrued over the last two decades to shorten the duration and improve the treatment outcomes. This multi-centric trial may generate crucial evidence with policy and practice implications in the treatment of TBM.

**Trial registration:**

Clinical Trial Registry India CTRI/2023/05/053314. Registered on 31 May 2023 (https://ctri.nic.in/Clinicaltrials/pmaindet2.php?EncHid=ODYzMzg=&Enc=&userName=CTRI/2023/05/053314). ClinicalTrials.gov NCT05917340. Registered on 6 August 2023 (https://classic.clinicaltrials.gov/ct2/show/NCT05917340).

**Protocol version:**

Version 1.3 dated 12 July 2023.

**Supplementary Information:**

The online version contains supplementary material available at 10.1186/s13063-024-08133-6.

## Background

Tuberculosis (TB) is a devastating disease that leads to significant morbidity and mortality. According to the Global TB report of World Health Organization (WHO), 7.5 million individuals were newly diagnosed with TB and 1.3 million succumbed to the disease [1]. Tuberculous meningitis (TBM) is a severe form of TB with high mortality and morbidity. Even though TBM contributes to 2% of all TB cases in low HIV prevalence settings, it accounts for 20 to 69% short-term mortality with standard anti-tuberculosis therapy (ATT) [2–5]. The case fatality ratio for central nervous system (CNS) TB is about 35% among all forms of TB in India [6]. A similar proportion of patients are also severely disabled with TBM [7]. A nationwide population-based cohort study with a 30-year follow-up revealed that TBM patients in Denmark had nearly twofold increased long-term risk of mortality compared to the general population [8]. The most common complications associated with TBM include hydrocephalus, hemorrhage, arachnoiditis, and formation of tuberculoma. The most commonly reported long-term neurological sequelae are motor deficits, epilepsy, cognitive impairment, and cranial nerve palsies [9, 10]. Stroke occurs in 15–57% of patients, of whom 20% suffer long-term neurological deficits [11].

The causes of mortality and morbidity in TBM are multifactorial, complicated by delayed presentations and poor diagnostic tests. The drug therapy used for TBM in adults and children also has significant limitations. The Infectious Disease Society of America, the American Thoracic Society, and the Centers for Disease Control and Prevention advise a regimen with 2-month intensive phase consisting of isoniazid, rifampicin, pyrazinamide, and ethambutol (HRZE) followed by 7 to 10 months of rifampicin and isoniazid (HR) in the continuation phase [12]. WHO recommends a 12-month regimen with 2 months of isoniazid, rifampicin, pyrazinamide, and ethambutol followed by 10 months of isoniazid and rifampicin in adults. The recent guidelines update supports a 6-month intensive regimen containing ethionamide (6HRZEto) for children and adolescents with TBM without evidence or suspicion of drug resistant tuberculosis (DRTB) [13]. Given the scarcity of available evidence concerning the optimal selection, dosage, and duration of antituberculous treatment (ATT), multiple regimens of varying duration are also being practiced. Additionally, the number of drugs administered differ during both the intensive and continuation phases. In addition to anti-TB drugs, there is also considerable variation in practice regarding the dose and duration of adjuvant therapy with corticosteroids and aspirin.

In designing this trial, our approach is guided by two principles. We wanted the intervention to reflect the improvements made in the field recently, including high-dose rifampicin and addition of fluoroquinolones and aspirin. We propose a novel clinical trial to demonstrate the efficacy of short course therapy compared to standard of care. The drugs and doses proposed in this trial have individually been shown to be beneficial and efficacious in the treatment of pulmonary tuberculosis and TB meningitis. However, they have not been evaluated for cumulative benefit. The INTENSE TBM trial that used high-dose rifampicin and linezolid evaluated mortality alone as an outcome and considered 30% reduction in mortality [14]. We hypothesize that intensified short course (6 months) ATT with high-dose rifampicin and moxifloxacin given along with corticosteroids and aspirin will significantly reduce mortality and disability in adults with TBM compared to 12 months of standard ATT regimen. We assume our study regimen will be superior to the control regimen in terms of reducing death or disability by at least 20%.

## Methods

This study protocol has been developed following the recommendations of SPIRIT (Standard Protocol Items: Recommendations for Interventional Trials) 2013 Statement, a guideline for a clinical trial protocol [15]. The SPIRIT checklist and flow diagram of this study protocol are shown in Additional file 1.

### Objectives

Our primary objective is to demonstrate the superiority of the intensified short course (6 months) ATT regimen containing high-dose rifampicin and moxifloxacin and standard ATT regimen (12 months) in reducing composite outcome of mortality or disability among adults with TBM. Secondary objectives are to assess the safety and tolerability of high-dose rifampicin and moxifloxacin when given daily for 8 weeks and pyrazinamide administration for 6 months. We also aim to compare the pharmacokinetic (PK) parameters of ATT in the serum and cerebrospinal fluid (CSF) between the three arms, and to compare the health-related quality of life between the intervention and control arms.

### Study design and setting

This is an open label, multi-center, parallel arms, randomized controlled superiority trial. The trial will be conducted across six sites located in different geographical regions in India. These sites are tertiary referral centers and well equipped with advanced laboratories, imaging facilities, and expertise to manage TBM and its complications. ICMR-National Institute for Research in Tuberculosis (ICMR-NIRT) will function as a primary sponsor and nodal coordinating center for the trial.

### Study population and eligibility criteria

Participants for the trial will be identified based on the Lancet consensus scoring system for the diagnosis of TBM [16]. This scoring system has 20 parameters divided into 4 categories: clinical, CSF, CNS imaging, and evidence of TB elsewhere. The maximum score is 20. If there is evidence of acid fast bacilli (AFB) in CSF smear, culture, or TB in histopathology of the brain or spinal cord, it is termed as a definite diagnosis of TBM. When the total score is > 10 with no CNS imaging, or > 12 with imaging, it is considered as probable diagnosis. A possible diagnosis is made with scores between 6 and 9 without imaging or 6–11 with imaging. All adults (> 18 years) with either definite, probable, or possible TBM as per the criteria will be eligible for the study. These participants should also be willing to undergo HIV test, consent, and adhere to trial procedures and follow-up schedule. They should also be residing within 100 km of the study sites. The female participants in the reproductive age group should agree to use effective barrier contraception during the period of the treatment.

We will exclude the patients if they have any of the following criteria: (1) known current/previous drug resistance to ATT such as rifampicin, isoniazid and luroquinolones; (2) concurrent or known diagnosis of any other meningitis such as bacterial, viral, and fungal; (3) currently having an uncontrolled cardiac arrhythmia or ECG abnormalities which are contradiction for the administration of moxifloxacin including prolonged QTc which is defined as > 450 ms in males and > 460 ms in females measured in lead II or V5 on a standard 12-lead ECG [17]; (4) has clinical icterus or hepatic impairment characterized by serum bilirubin level above the normal laboratory reference range with abnormal liver enzymes, or isolated alanine aminotransferase and/ or aspartate aminotransferase levels above five times the upper limit of the normal laboratory reference range [18]; (5) previous history of ATT, if any, should not exceed 1 month in the past and not more than 7 days in the preceding 1 month; (6) pregnant or lactating women; (7) rapid clinical deterioration or very sick and moribund during the screening process, renal failure, liver disease, or any condition (social or medical) that in the opinion of the investigator would make trial participation unreliable or unsafe; and (8) has a known allergy to any of the drugs proposed to be used in the trial regimen.

### Randomization

Computer generated list of random numbers using permuted block randomization will be created and uploaded in RedCap data base.When all the screening results are available and within the normal range, the patient will be randomly allocated in a ratio of 1:1:1 to one of the treatment groups. The study sites will be required to ensure inclusion and exclusion criteria for each patient prior to enrolment and enter the data in the baseline case report form with the eligibility criteria in RedCap data base. Upon verification of the case report form and the eligibility criteria, the ICMR-NIRT statisticians (randomization team) will approve for randomization and site investigators can proceed for online randomization in Redcap.

 Allocation concealment will be ensured, as the randomization team will not approve the randomization until the patient has been recruited into the trial, which takes place after all baseline assessments are completed. The intervention will be administered by the site investigators who will also be treating physicians.

### Treatment allocation

#### Arm 1 (intensified ATT with aspirin)

**Fig. 1:**
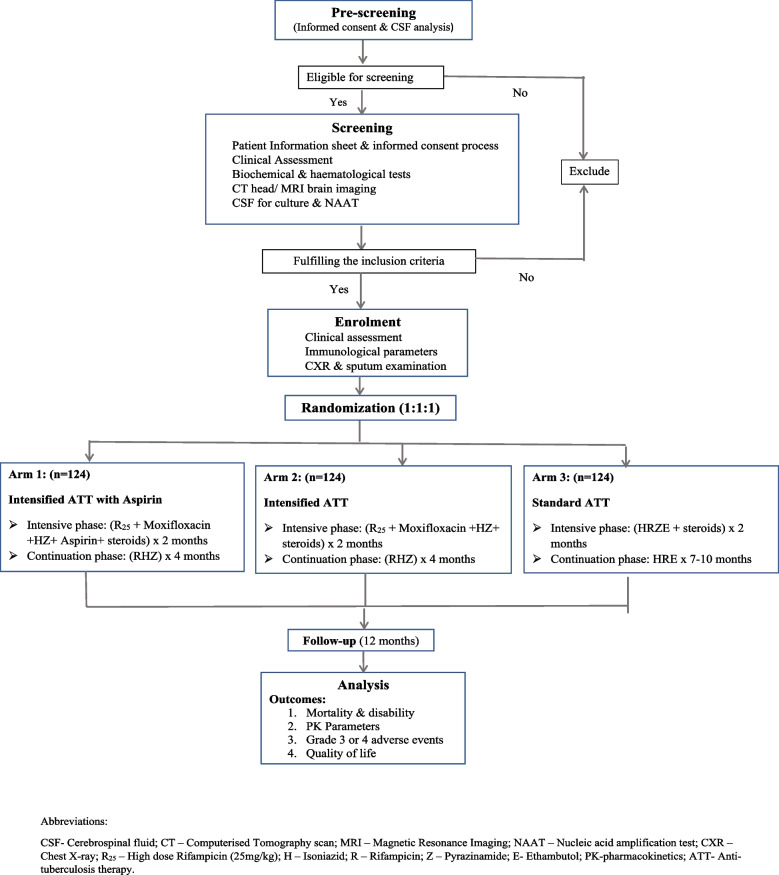
Study schema

Participants in this arm will receive a high dose of rifampicin 25 mg/kg once daily and moxifloxacin (400 mg once daily) along with isoniazid and pyrazinamide for 2 months. Steroids will be administered to participants with MRC grades 1 and 2 for a duration of 6 weeks, while participants in grade 3 will be treated for 8 weeks. This regimen will also be intensified with 150 mg of aspirin once daily. Intensive phase will be followed by standard doses of rifampicin, isoniazid, and pyrazinamide for the next 4 months in the continuation phase (Fig. 1).

#### Arm 2 (intensified ATT without aspirin)

Participants in this arm will receive intensified ATT regimen and steroids like arm 1 but without aspirin in the intensive phase and standard doses of isoniazid, rifampicin, and pyrazinamide for the next 4 months in the continuation phase.

#### Arm 3 (control arm)

The control arm will receive treatment according to the standard national guidelines for TBM. They will receive a first-line regimen consisting of 2 months of isoniazid, rifampicin, ethambutol, and pyrazinamide followed by 10 months of isoniazid, rifampicin, and ethambutol daily in a fixed-dose combination (FDC) as per the various weight bands in National TB Elimination Programme of India. Participants will also receive steroids as in the other two arms.

### Recruitment

It is estimated that each collaborating institutes treat 80–120 patients with TBM each year. Our study team will work with departments of emergency medicine, internal medicine, and neurology. Any patient with symptoms and signs suggestive of TBM and undergoing lumbar puncture will be pre-screened for the trial after obtaining written informed consent. During pre-screening, CSF collected will be sent for gram staining, bacterial culture and sensitivity, AFB smear, MGIT, Gene Xpert/Xpert Ultra, Truenat, and any other laboratory studies as per the treating clinician’s advice. Once the patient is found to be diagnosed with TBM after pre-screening, screening assessments will be done after obtaining written informed consent explaining the details of the trial with participant information sheet. Written informed consent will be taken by the trial nurse. Participants information sheet and informed consent has been given as Additional file 2.

### Participant timelines and follow-up

In this assessment, demographic details, medical history, clinical examination, neurological assessment including Modified Rankin Score (MRS) and Glasgow Coma Scale (GCS), sociological assessment for compliance to trial procedures, and all other study procedures will be done. Blood will be collected for hematological (complete blood count—CBC) and biochemical tests (sugar, HbA1C, liver, and renal function tests), HBsAg, and HIV. Patients will also undergo CT or MRI imaging of the brain. We will also collect sputum for CBNAAT and AFB culture and do chest X-ray to rule out pulmonary TB. All eligible patients will be enrolled into the trial. At the time of enrolment, blood will be collected for immunological biomarkers (CRP, TNF-α, monocyte and dendritic cell functions, and other cytokines).

During the study period, patients will be followed as per the study schedule attached (Table 1). After admission to the study and treatment allocation, participants will be followed up every week for the first 2 months and monthly till the completion of the treatment. They will undergo clinical examination including detailed neurological examination and adverse event (AE) monitoring. After treatment completion, all participants will be followed up once in 3 months for a period of 12 months. Participants in the study arms I and II will complete 12 months of post-treatment follow-up at the 18th month study visit and participants in the control arm will complete their post-treatment follow-up at the 24th month study visit.

**Table 1:** Study schedule

S No	Investigations	Pre-screening	Screening	Enrolment	W1	W2	W3	W4	W5	W6	W7	W8	M3	M4	M5	M6	M7	M8	M9	M10	M11	M12	M15	M^a^18	M21	M24
1	Screening consent		✓																							
2	Assess eligibility		✓	✓																						
3	Informed consent			✓																						
4	History and clinical examination		✓	✓	✓	✓	✓	✓	✓	✓	✓	✓	✓	✓	✓	✓	✓	✓	✓	✓	✓	✓	✓	✓	✓	✓
5	GCS		✓	✓	✓	✓	✓	✓	✓	✓	✓	✓	✓	✓	✓	✓			✓			✓	✓	✓	✓	✓
6	Modified Rankin’s score		✓	✓	✓			✓				✓		✓		✓			✓			✓	✓	✓	✓	✓
7	Sputum smear and MGIT^b^		✓																			✓		✓		
8	CSF analysis for AFB smear, MGIT, Xpert, and to rule out other etiology as appropriate	✓																								
19	CSF cytology, biochemistry,	✓																								
10	HIV		✓																							
11	Urine pregnancy test^c^		✓					✓				✓	✓	✓	✓	✓	✓	✓	✓	✓	✓	✓				
12	Hepatitis B and C		✓																							
13	LFT		✓			✓		✓		✓		✓		✓		✓			✓			✓				
14	RFT		✓			✓		✓		✓		✓		✓		✓			✓			✓				
15	Uric acid					✓		✓		✓		✓		✓		✓										
16	HbA1C (only for diabetics)		✓										✓			✓			✓			✓				
17	Hematology		✓					✓				✓				✓			✓			✓				
18	CD4 count if HIV positive		✓					✓				✓				✓			✓			✓				
19	Imaging (CT/MRI)		✓													✓										
20	CXR^b^		✓																							
21	ECG in 12 leads		✓					✓				✓				✓										
22	Intensive PK Serum^d^					✓																				
23	Sparse PK Serum					✓		✓				✓														
24	CSF PK (single time point)^d^																									
25	Adverse event assessment			✓		✓	✓	✓	✓	✓	✓	✓	✓	✓	✓	✓	✓	✓	✓	✓	✓	✓				
26	Storage of plasma and serum sample		✓					✓				✓				✓			✓			✓				
27	Neurological assessment			✓	✓	✓	✓	✓	✓	✓	✓	✓				✓			✓			✓		✓		✓
28	Quality of life assessment			✓												✓						✓		✓		✓

^a^Participants in the intervention arm will complete 12 months of post-treatment follow-up at 18th month study visit and participants in the control arm will complete their post-treatment follow up at 24th month study visit

^b^At baseline and when symptomatic

^c^Urine pregnancy test in women with reproductive potential

^d^Intensive PK serum and CSF to be planned between weeks 1 and 2

### Pharmacokinetics study

A full pharmacokinetic (PK) study of ATT will be carried out in 15 patients per arm per site in two sites any time between first and second week study visits. On the day of PK, the patient will be requested to be on an empty stomach. A sample of blood (2 ml) will be collected (0 h). The study drugs will be administered under direct supervision and the time of drug administration will be noted. Thereafter, 2 ml of blood will be collected at 2, 4, 6, 8, and 12 h. A single CSF sample for PK (preferably 2nd hour after ATT administration) will be also be collected. The same patients will undergo a sparse plasma PK (0, 2, and 4 h) at the end of the fourth and eighth week.

### Patient compliance

The drugs will be administered under supervision (except on Sundays) to ensure compliance. The importance of adherence to the treatment schedule will be reinforced at each visit. The cause for non-adherence will be identified. If the patient misses drug doses in the intensive phase (IP), it will be compensated at the end of the intensive phase. The compensation will be given for a maximum period of 15 days to complete 60 doses of the assigned regimen in the IP. Similarly, the missed doses in the continuation phase (CP) will be compensated at the end of CP so as to complete 120 doses in CP. Patients who do not attend treatment continuously for more than a month will be considered as lost to follow-up. For these patients, a minimum of two rounds of three attempts to contact them (telephonic/home visit) will be made. If these attempts are unsuccessful, then the participant will be considered lost to follow-up.

### Concomitant medications

Concomitant antibiotic treatments of any kind are discouraged during the period of study drug administration unless absolutely indicated. During the course of the trial, a short course (< 2 weeks) of antimicrobial therapy may be permitted for concurrent illnesses. Short courses of antibiotic therapy (< 2 weeks) with drugs not indicated for the treatment of TB will be limited and the illness for which they are prescribed will be recorded. The following agents used to treat M. tb infections will not be used during the trial: streptomycin, thiacetazone, PAS, dapsone, amoxicillin clavulanic acid/clavulanate, clofazimine, capreomycin, any oxazolidinone antibiotic (e.g., linezolid), ofloxacin, levofloxacin, bedaquiline, pretomanind, and delamanid.

### Adverse event (AE) management

The participants will be closely monitored during the scheduled study visits. AEs will be collected from the initiation of treatment regimen onwards. All serious adverse events (SAEs) occurring during the trial period must be reported to the Institutional Ethics Committee (IEC) i.e., within 24 h of the investigator becoming aware of the event. They will also be reported to the sponsor and the competent authorities within the specified period of time as defined in New Drugs and Clinical trials Act 2019. The AEs will be graded as per DAIDS grading Table 2017 and managed as per protocol especially for grade III and grade IV events, hepatotoxicity, and cardiovascular system events [19].

### Criteria for discontinuing interventions

*Grade 2 toxicities:* For grade 2 toxicities, the patient will be followed more carefully, with additional laboratory and/or clinic visits as necessary, and the study drugs temporarily held at the investigator's discretion.

*Grade 3 toxicities:* For any grade 3 toxicity that in the investigator’s judgment is due to study drug(s), the causative study drug(s) will be held. The clinician will rule out other possible causes of the symptoms before discontinuing study medication. When possible, concomitant medications will be held first at the discretion of the investigator if he/she suspects they are contributing to the toxicity. Depending on the nature and severity of the toxicity, the degree to which it resolves, and/or the emergence of alternative explanations for the toxicity or the participant’s deterioration, the study drugs(s) may be restarted at the discretion of the investigator.

*Grade 4 toxicities:* Study drug will be permanently discontinued in any participant with grade 4 renal, hepatic, cardiac, or hematological toxicity.

For all toxicities that are treatment-emergent and require the study therapy to be temporarily or permanently discontinued, relevant clinical and laboratory tests will be obtained as clinically indicated and repeated as needed until final resolution or stabilization of the toxicity.

### Sample size

The trial is powered to assess the superiority of intensified regimen (arm 1) compared to control regimen (arm 3). A systematic review and meta-analysis on treatment outcomes in TBM done by Stadelman et al. reported the proportion of mortality and disability after treatment is 24% and 32%, respectively [7]. The proportion of composite outcome (death or disability) is 56%. We assume a 20% reduction of composite outcome with our intervention (i.e., from 56 to 36%). We used the formula for Z test for comparing proportion between two groups (arm 1 and arm 3). Hence, to study the impact of “Intervention” compared to “Standard care” in reducing the composite outcome (death or disability), expecting a minimum difference of 20% between intervention arm with aspirin (arm 1) and control arm (arm 3), assuming 85% power of the study, 5% level of significance, 2-sided significance test; the required sample size was 112 patients in each arm. Considering 10% lost to follow-up, the total sample size was calculated as 372 with 124 in each arm. Previous clinical trials in TBM have evaluated mortality as an outcome and considered 30% reduction [14]. Since we propose to evaluate composite outcome of death or disability, we have assumed a conservative effect size of 20% reduction. The 10% loss to follow-up is assumed based on the previous TBM trials as well as randomized controlled trials conducted at ICMR-NIRT, Chennai [20, 21].

### Data management

#### Data collection

Data which includes demographic, clinical, and laboratory information will be collected in case report forms (CRF). Data will be entered in the electronic data base after checking for completeness as per the detailed SOP and transmitted to the sponsor through a secure, web-based software system. The study site PI shall be responsible for the data quality and management. A central data management team will periodically review the quality of the data. An alphanumeric ID will be assigned to each participant to preserve personal information and contacts.

### Data analysis

Participants who have completed 80% of the prescribed treatment will be considered for per protocol (PP) analysis and all the patients who were randomized will be taken for intention-to-treat analysis. Modified intent-to-treat (mITT) will be performed after excluding: (a) patients with DR TBM detected after randomization; (b) diagnosed to have any other meningitis such as bacterial, viral, and fungal after randomization; (c) patients refused to be part of the trial when the initial consent was obtained from legally authorized representative; and (d) receiving less than 2 months of administration of the randomized study drugs for reasons other than death.

An interim analysis is planned at two time points during the study period, viz. after 33% and 66% of the enrolled patients have completed 8-week treatment. In addition, the interim analysis may be done if the frequency of reported serious adverse events is greater than anticipated. Reports will be provided to the DSMB at these time points.

Baseline variables will be summarized between the groups using appropriate summary statistics based on the level of measurement. The time to composite outcome will be estimated using Kaplan–Meier survival estimate and it will be compared between the three arms using Log rank test. The factors associated with time to death or disability will be assessed using Cox proportional hazards regression analysis. This is a superiority trial and we will perform two-sided tests and use type I error alpha value of 5% to claim superiority between two groups.

The analysis for the primary outcome will be stratified based on HIV status and baseline severity of the disease measured by MRC. Sensitivity analysis will also be done including only definite and probable cases of TBM. The Cox proportional regression will be summarized using hazard ratio and 95% confidence interval. Extended Cox regression will be used in lieu of proportion Cox regression if the proportional hazard assumption is violated. The AE data will be summarized using frequency and percentage and compared between the regimens using Chi-square test. In addition, count regression models will be used to compare the number of AEs between the regimens after adjusting for other variables. Maximum plasma concentration (*C*max), time for maximal concentration (*T*max), and area under the curve (AUC) will be measured and correlated with treatment outcomes and occurrence of adverse events using log binomial regression model. Influence of PK parameters on treatment outcome will be identified using multivariable regression analysis. The quality of life will be summarized using mean and standard deviation. The variable will be compared between the regimens using ANOVA or the non-parametric alternative, Kruskal–Wallis test.

We will deal the missing data using multiple imputation technique depending on the missing data mechanism.

### Trial monitoring

A Data Safety and Monitoring board (DSMB) will review data and monitor progress of the trial and to detect evidence of early safety issues for the participants. The DSMB will consist of TB clinicians, a pharmacokinetic specialist, and an independent biostatistician. The trial will be monitored by an independent trial monitor to verify the accuracy, completeness of the trial data and the conduct of the trial is in compliance with the applicable regulatory requirements and Good Clinical Practice (GCP). The IEC of ICMR- NIRT and all the participating institutes will also oversee the conduct of the trial.

### Outcome measures

Our primary outcome is mortality or disability which will be measured between inclusion and month 12 and month 24, i.e., time from randomization to death or disability during the follow-up period. All-cause mortality also will be considered as an outcome in the study. Survivors will be censored at the date they were last known to be alive (i.e., date of last follow-up visit, loss to follow-up or withdrawal). All-cause mortality rate will be measured in proportion and disability will be measured by modified Rankin scale. We will classify the score as “no disability” (a score of 0), “mild to moderate disability” (score of 1–2), and “severe disability” (3–5).

Grade 3 or 4 AEs will be measured in proportion according to Division of AIDS (DAIDS) criteria at the end of 12 months [18]. The DAIDS criterion categorizes adverse events as “mild events” (grade 1), “moderate events” (grade 2), “severe events” (grade 3), and “potentially life threatening events” (grade 4). Pharmacokinetic outcome measures will be plasma concentration time-curve during the dosing interval (AUC), peak plasma concentration (*C*_max_), and CSF concentration. Time to reach maximum plasma concentration (*T*_max_) of the study drugs will be measured up to week 8. Quality of life at baseline and 6 months, 12 months, and 24 months will be assessed in mean and standard deviation using a WHO Short form-36 (SF-36) questionnaire. It is a commonly used generic health-related quality of life (QoL) instrument which consists of 36 questions measuring health in different dimensions covering physical, mental, and social well-being of the patients. SF-36 was adapted and translated into several language and its validity and reliability were already established in India in TB context [22, 23].

### Ethical considerations

This study will be conducted in accordance and compliance with Indian standards of Good Clinical Practice (Indian GCP), ICH-GCP, Declaration of Helsinki (WMA, 2013), CDSCO guidelines, Ethical Guidelines for Biomedical Research on Human Participants 2017 (Indian Council of Medical Research, New Delhi), applicable Government of India Regulations, and research policies and procedures of the institute. This trial has been registered prospectively with Clinical Trial Registry of India and ClinicalTrials.gov with the registration numbers CTRI/2023/05/053314 and NCT05917340, respectively. All participants during pre-screening and screening will be given participant’s information sheet and obtained written informed consent. Participants will be informed that their participation is voluntary and that they may withdraw from the trial at any time. If a participant is unable to give the consent participant due to altered sensorium or severely illness, the consent will be taken by the legally acceptable/authorized representative (LAR) and a re-consent will be taken once the participant has ability to understand the study procedure and in a position to give consent. The confidentiality of the participant’s details will be ensured. Consent will also be obtained for use of biological samples in the future. The results of trial will be published in peer-reviewed medical journals and disseminated at national and international conferences. ICJME criteria will be adhered while defining the authorship. Any modifications, deviation, and amendments to the protocol will be intimated to the ethics committees of ICMR-NIRT and respective sites time-to-time. The informed consent form will be revised as per protocol amendments and re-consent will be obtained will be obtained from the participants after sharing the relevant information.

### Confidentiality

The processing of personal data in this trial will be limited to those data that are reasonably necessary to investigate the anti-bacterial activity, safety, and tolerability of the investigational product used in this trial. These data will be processed with adequate precautions to ensure confidentiality.

The study participant will be informed during the informed consent process that—the investigators, monitors, the auditors, the IEC, and the regulatory authorities will be granted direct access to the participant’s original medical records for verification of clinical trial procedures and/or data, without violating the confidentiality of the participant, to the extent permitted by the applicable laws and regulations and that, by signing a written informed consent form, the participant or the participant’s legally acceptable representative is authorizing such access. The participants will not be identified in any presentations or publications based on the results of this research. The sponsor or its representatives whose responsibilities require access to personal data are obliged to keep the identity of trial participants confidential. This confidentiality will be maintained throughout the complete data processing. We do not have plans of granting access to full protocol, participant level data set, or statistical code at the moment. However, we may do so once the findings of the trial are disseminated.

### Ancillary and post-trial care

Participants enrolled into the trial will be covered by insurance for non-negligent harm associated with the protocol. This will include cover for additional health care, compensation for trial-related injury, and death. ICMR-NIRT will facilitate this compensation through insurance based on the recommendation of the ethics committee.

## Discussion

Our trial is one of the attempts to reduce the high morbidity and mortality associated with TBM. High-dose rifampicin has been proved to be efficacious in patients with pulmonary TB. A meta-analysis of eight studies reporting on higher doses of R (13 to 35 mg/kg) in patients with pulmonary TB demonstrated accentuated sputum culture conversion as compared to the standard-dose group. The authors also reported no increased adverse events in the high dose rifampicin group [24]. Rifampicin in the current dosages and ethambutol used in the current regimen for the management of TBM have limited CNS penetration. In patients with TBM, high doses of rifampicin are associated with increased CSF exposure, improved clinical outcomes without increased grade III or IV adverse events [25–27].

Ruslami et al. showed a 50% reduction in mortality compared to the standard oral dose (10 mg/kg) when participants were given a higher dose of intravenous rifampicin (13 mg/kg/day) in adults with TBM in Indonesia. The trial also compared ethambutol (750 mg) with moxifloxacin (400 mg vs 800 mg). Hepatotoxicity of all grades was equally distributed across the groups [25]. Higher dose of rifampicin did not increase drug-related AEs and improved the survival of patients [25]. Dian and colleagues administered three different oral doses of rifampicin (10 mg/kg vs 20 mg/kg vs 30 mg/kg) along with isoniazid, pyrazinamide, and ethambutol and compared with standard TB regimen in adults with TBM. They also demonstrated lower incidence of 6-month mortality without increase in the incidence of grade 3 and 4 AEs [27]. High-dose intravenous and oral rifampicin were found to be safe in the trial. Adults from Uganda who were suspected of having TBM were randomized to receive high-dose oral rifampicin (PO-35, 35 mg/kg/day), intravenous rifampicin (IV-20, 20 mg/kg/day), or standard-of-care control (PO-10, rifampicin 10 mg/kg/day) along with isoniazid, pyrazinamide, and ethambutol. The composite safety endpoint did not differ between the three arms (*P* = 0.342). Rifampicin exposure in the CSF was approximately eightfold higher in the oral high dose arm (35 mg/kg) than the standard of care with no additional toxicity [28].

Charlie et al. conducted a meta-analysis of five trials that included 1028 participants with TBM. They reported that though high-dose of rifampicin did not reduce mortality, there was no significant increase in the risk of grade 3–5 adverse events. The authors recommended that future trials are required to conclude on the optimal dose of rifampicin and the efficacy of such a high dose in reducing mortality among patients with TBM [29]. In summary, doses up to 25 mg/kg to 35 mg/kg appear safe in studies from Africa and Asia, but large-scale studies evaluating clinical efficacy are needed.

Thwaites et al. demonstrated better CSF penetration among TBM patients when given ciprofloxacin (750 mg every 12 h) or levofloxacin (500 mg every 12 h) or gatifloxacin (400 mg every 12 h) in addition to conventional four-drug regimen. Among the agents, CSF penetration was greatest with levofloxacin [30]. Allfennar and colleagues concluded that though moxifloxacin enters the CSF at the same rate as levofloxacin, it accumulates well in the CSF and thus has the theoretical advantage of greater in vitro activity against M. tb than levofloxacin [31].

Current WHO guidelines 2022 recommend 4-month moxifloxacin-containing regimen with rifapentine and INH for 4 months along with pyrazinamide in the initial 2 months for pulmonary TB. However, this regimen is not recommended for TBM at this juncture due to a lack of evidence [32]. Pyrazinamide is unique as it works against the non-replicating persisters and has a sterilizing activity and good CNS penetration. One of the earlier clinical observations from India using 1500 mg of pyrazinamide along with isoniazid 300 mg and rifampicin 450 mg. Pyrazinamide was continued after the intensive phase, and it was observed that no patients presented with new symptoms suggesting focal deficit and/or increased intracranial pressure during follow-up [33]. Another prospective cohort study from South Africa also confirmed similar findings. However, this has not been studied in a RCT yet [34].

While the mortality due to TBM among hospitalized patients was 42%, disability among them has been reported as 32% leading to poor quality of life among survivors [7, 35]. This highlights the importance of measuring disability as a primary outcome in addition to mortality. Evidence suggests that aspirin reduces the new-onset stroke, a major cause of disability in patients with TBM by significantly reducing perivascular inflammation. According to a meta-analysis with 365 participants from three randomized trials, aspirin effectively decreased the incidence of new-onset stroke with a number needed to treat of 10 [36]. The doses of aspirin used in these trials also varied widely. A trial from India showed lower dose aspirin (150 mg/day) when used along with steroids prevented new infarcts and reduced strokes and mortality [37]. In contrast, Schoeman et al. demonstrated in an open-label trial that aspirin use, regardless of dosage, did not significantly reduce mortality and morbidity (hemiparesis) [38]. Hence, it is vital to prove the efficacy of aspirin in reducing the stroke with an adequate dose for an appropriate period. Thus, we propose 150 mg of aspirin to all TBM patients in one of the intervention arms for 2 months. Thwaites et al. showed in a large RCT, that while there is a significant reduction in mortality in the steroid group, the proportion of patients with severe disability was higher (18.2 vs 13.2%) [39]. An almost-quarter reduction in mortality was observed with the use of steroids, according to a systematic review and meta-analysis comprising 9 trials involving 1337 participants (RR-0.75, 95% CI: 0.65–0.87). Additionally, the analysis concluded that steroid use might have minimal or no impact on the neurological deficit [40]. The role of adjunctive strategies like aspirin on disability needs further study.

In conclusion, the mortality and morbidity with the currently used regimens are very high despite current treatment regimens. Studies evaluating shorter, more efficacious regimens are urgently needed. We optimize currently available common drugs in this RCT to impact morbidity-free survival in tuberculous meningitis.

### Trial status

The trial has not started recruiting the participants yet. It is anticipated that this trial will commence on May 15, 2024, and close by October 15, 2027. This version refers to Version 1.3 of the approved protocol dated July 12, 2023.

### Supplementary Information

**Additional file 1.** SPIRIT checklist.

**Additional file 2.** Participants information sheet and informed consent.

## Data Availability

Not applicable.
