# Supplementary material for: Comparative evaluation of intensified short course regimen and standard regimen for adults TB meningitis: a protocol for an open label, multi-center, parallel arms, randomized controlled superiority trial (INSHORT trial)
Source: Trials. 2024 May 2;25:294. doi: 10.1186/s13063-024-08133-6 (PMC11064413; doi:10.1186/s13063-024-08133-6)
Supplement: Supplementary file 2 — Additional file 2. Participants information sheet and informed consent. [file 13063_2024_8133_MOESM2_ESM.docx]

**Participant information sheet**

| Title of the study | **Comparative evaluation of intensified short course regimen and standard regimen for adults TB meningitis : an open label randomized controlled trial (INSHORT trial)** |
| --- | --- |
| Version, date of information sheet | Version 1.2, dated 12 July 2023 |
| Principal Investigator | Dr Leeberk Raja.I, Scientist ‘E’,  ICMR- National Institute for Research in Tuberculosis,  No:1, Mayor Sathyamoorthy road,  Chetpet, Chennai – 31  Contact number – 044-28369500 |

Dear Participant,

You are invited to be part of a research study being conducted by ICMR- NIRT, Chennai. Please find below the relevant information regarding the study for you to make a voluntary decision regarding your participation in the study. If you have any questions about this study, please feel free to ask before deciding to participate in the study.

**1.Basic information**

We have found from the tests done in this hospital, you have been diagnosed with tuberculous meningitis (TBM), a form of tuberculosis (TB) that affects the brain. TB is an infectious disease that spreads from one person to another when he/ she coughs or sneezes. TB is a curable disease if you take the prescribed treatment regularly. When TB affects the brain it’s called as tuberculous meningitis (TBM). The current treatment for TBM is given for 9-12 months. However, this treatment regimen has higher death and disability rates. It has been shown in the recent research that by modifying the treatment with increasing the dose of already used drugs and adding few additional drugs can be more effective than the regimen that is currently being used. We believe that the modified regimen when given for 6 months could reduce the death and disability. We are planning to recruit patients with TBM and study the effect of this modified regimen across India. We will compare this modified treatment regimen given for 6 months with standard treatment given for 12 months. We will follow up all these patients for 1 year after completion of treatment to see if there is a recurrence of the disease and to identify any new complications due to the disease and the modified regimen.

**2.Information about the trial**

You are being asked to take part in this research study because you are diagnosed with TBM). The infection is in your brain and can be life-threatening if left untreated. It is generally treated with a course of four or five anti TB drugs for a period of 9 to 12 months. The current research suggests that even with drug treatment, death or long-term complications such as stroke, disability may occur in TBM.

The purpose of this study is to find out if reducing the treatment duration to 6 months with increasing the dose of a standard TB drug and/or changing one of the drugs and adding few more drugs will reduce deaths and long-term complications. Participants will be assigned by chance to one of three study groups:

1. The first group will receive high dose of rifampicin, moxifloxacin, isoniazid, pyrazinamide, aspirin along with steroids for 2 months. In the next 4 months, we will substitute ethambutol with pyrazinamide. You will receive rifampicin, isoniazid and pyrazinamide for 4 months.
2. The second group will receive high dose of rifampicin, moxifloxacin, isoniazid, pyrazinamide along with steroids for 2 months. This group will not receive aspirin. In the next 4 months, we will substitute ethambutol with pyrazinamide. You will receive rifampicin, isoniazid and pyrazinamide for 4 months
3. The third group will receive standard drug treatment with isoniazid, rifampicin, pyrazinamide, and ethambutol along with steroids for 2 months followed by rifampicin, isoniazid, and ethambutol at standard doses for 10 months.

This newer regimen has been decided based on evidence from multiple studies done in adults with TBM meningitis. Adults ≥ 18 years of age diagnosed with possible, probable or confirmed TBM meningitis may join this study. We are planning to recruit 372 adults with TBM from different sites across India.

**3.Expected duration of participation**

You will be in this research study for a duration of 18 months or 24 months.

**4.Description of procedures to be followed**

If you agree to take part in this study, before the administration of the drugs you will go through the following procedures and tests.

- Medical history information will be collected from your medical records and by talking to you.
- A study doctor will examine you and we will measure your weight, height, temperature, and or additional measurements that will not cause you any discomfort.
- We will ask you about medications that you are currently taking or has taken in the last 14 days.
- We will collect about 2 tsp (5- 10 ml) of blood to test your liver, kidney, blood counts, and for additional tests .
- We will also do a test for HIV (Human Immunodeficiency Virus) infection after the counselling. Testing for HIV is recommended for everyone with TB and is needed for this study. Please note that the results of these tests will be kept confidential at all times.
- You will have a chest x-ray. This is standard of care because often the bacteria that causes TB meningitis is also found in the lungs.
- You may have to undergo a computerized tomography (CT) scan or MRI of the brain which will help in diagnosing TBM. The CT and MRI are sometimes done as standard of care and may not be repeated if they have already been done with in the last two weeks.
- We will also do an ECG and assess your quality of life by asking a few questions.
- You will have a lumbar puncture (spinal tap). This procedure involves taking a small sample of cerebrospinal fluid (CSF) for examination. CSF is a clear, colourless liquid that delivers nutrients and “cushions” the brain or spinal cord. During a lumbar puncture, you will be positioned with your back curved out and a needle is carefully inserted into the lower spine to collect the CSF sample. This is standard of care for diagnosing and managing TB meningitis. The CSF sample will be sent to a laboratory to look for the organism that causes disease including TB meningitis.

After the initial procedures and test if you are found to be eligible and willing for the study, you will be randomly assigned to one of three study treatment groups for TBM. The treatment will be chosen by chance. You and the study staff will know which group you are in and all study medicines used for treatment in this study will be given once daily. All the drugs will be given through mouth (orally) except steroids which will be given as intra-venous injection initially and switched over to oral dose later. The dose of the steroids will be reduced over 8 weeks. You will get one of the following:

| **Group – 1 (Intensified with aspirin)** | **Group- 2 (Intensified without aspsirin)** | **Group -3 (Control group)** |
| --- | --- | --- |
| ***2 months*** | ***2 months*** | ***2 months*** |
| - Rifampicin: 25 mg/kg - Isoniazid :10mg/kg - Pyrazinamide: 35mg/kg - Moxifloxacin :400mg - Aspirin : 150mg - Prednisolone or Dexamethasone – (0.3 mg/Kg or 0.4 mg/kg depending on the severity). | - Rifampicin: 25 mg/kg - Isoniazid :10mg/kg - Pyrazinamide: 35mg/kg - Moxifloxacin :400mg - Prednisolone or Dexamethasone – (0.3 mg/Kg or 0.4 mg/kg depending on the severity). | - Rifampicin:10/mg/kg - Isoniazid: 10 mg/kg) - Pyrazinamide:35mg/kg - Ethambutol: 20 mg/kg - Prednisolone or dexamethasone - (0.3 mg/Kg or 0.4 mg/kg depending on the severity). |
| ***4 months*** | ***4 months*** | ***10 months*** |
| - Rifampicin: 15 mg/kg - Isoniazid :10mg/kg - Pyrazinamide:35mg/kg | - Rifampicin: 15 mg/kg - Isoniazid :10mg/kg - Pyrazinamide:35mg/kg | - Isoniazid: 10 mg/kg - Ethambutol: 20 mg/kg - Rifampicin: 15 mg/kg |

***Follow- up visits***

During the study period, patients will be followed up during the treatment and one year after the treatment. If you are part of the study, we will monitor your progress and response to the treatment every week for the first 2 months and monthly till the completion of the treatment. You will undergo the following

- Medical history information will be collected from your medical records and by talking to you.
- A study doctor will examine you and we will measure your weight, height, temperature, and or additional measurements that will not cause you any discomfort.
- If you have cough with sputum, we will collect sputum for further tests
- If you are a female patient and in the reproductive age group you will be required to have a urine pregnancy test before treatment initiation and during the treatment period, and should be willing to adopt effective birth control measures for the treatment period
- We will collect 2 teaspoon of blood (5-10 ml) to monitor your kidney and liver functions and blood counts. We will perform kidney and liver function tests once in two week the first 8 weeks and then at 4,6,9,12 months in both treatment groups. We will check your blood counts and sugar once in a month.
- We will also do CT/MRI at 6^th^ month for patients in the both groups.
- We will do an ECG at the end of month 1, 2 and 6 months for the both groups
- We will also assess your quality of life at the end of the treatment and at month 6 and 12 months after the completion of the treatment in both the groups.
- We will store blood and serum storage at weeks 1, 4, 8, and at 6^th^, 9^th^ and 12^th^ month for further analysis and future research that may be related to infection of the central nervous system.

### Drug level visits (PK)

If willing, some of you may be asked to come for additional visits to do few more tests to measure the drug level in blood and CSF. This test helps to figure out if you are receiving enough dosages of the drugs. If you are taking part in this test, you will be asked to come on an empty stomach . There will be 6 blood draws over 12 hours to measure the amounts of drugs in your blood over time. Breakfast will be provided by the study team after taking the 1^st^ hour blood sample. We will collect about 2 teaspoons (10 ml) of blood before you take the study drugs and then later 2,4,6,8 and 12 hours. We will do this drug level test between week-1 and week-2 after the treatment in 60 willing patients .We will collect a single CSF sample on that day preferably coinciding with one blood draw time point. The drug levels will be measured in the collected CSF. Any remaining CSF from the lumbar puncture and blood will be stored for retesting or future research if needed. The same patients will go for drug level testing at the end of 4^th^ and 8^th^ week of treatment. During this time,

we will collect about 2 teaspoons (10 ml) of blood before you take the study drugs and then later 2,4 hours. Your specimens will be labelled using unique identification number and stored for testing.

***Post treatment follow up visits***

After the end of treatment, you will be followed up once in three months for a period of 12 months. If you are part of the group-1, you will complete 12 months of post-treatment follow-up at the 18th-month study visit and in the group-2, you will complete post-treatment follow-up at the 24th-month study visit.

- Medical history information will be collected from your medical records and by talking to you.
- A study doctor will examine you and we will measure your weight, height, temperature, and or additional measurements that will not cause you any discomfort.
- If you have any symptoms suggestive TB, we will do further tests.

**5.Foreseeable risks/discomfort**

**Drugs:**

The drugs used are generally known to be safe. The most common side effects are abdominal (stomach) problems, such as vomiting or diarrhoea. The drugs given for the research and not part of standard care are moxifloxacin and high dose rifampicin and aspirin. The most common side effects of those study drugs are mentioned below and there are few more uncommon side effects in addition to the list mentioned here.

***Rifampicin***

The most common side effects of rifampicin are rash, nausea, vomiting, liver problems. Body fluids such as sweat or urine may change color to orange or red.

***Moxifloxacin***

Some of the known side effects are mental disturbance, dizziness, fainting, irregular or fast heartbeat, convulsions, and rhythm changes in the heart.

***Aspirin***

Nausea, vomiting, diarrhea and increased tendency of bleeding in the internal organs.

***Sterioid***

Bleeding from stomach, elevation of sugars and blood pressure, reduction in potassium in blood, mental disturbances

**Blood Draws:** You will need to provide blood samples, for various blood tests as listed above. Drawing of blood may cause certain discomfort and pain. The risks of drawing blood may include rarely fainting and/or bruising. Very rarely, there may be a small blood clot or infection at the site of needle puncture (where needle goes in). For your protection, these procedures will be carried out under sterile (germ free) conditions and a trained phlebotomist will draw the blood. Standard of care will be given to you in case any of these are encountered.

**Lumbar Puncture:** The risks of lumbar punctures are minimal but include headache. It’s recommended that patients lie down for a few hours after the test and drink plenty of fluids to prevent headaches. The procedure is usually not very painful, but momentary twinges of pain may be felt if the needle brushes against nerve tissues.

**X-ray:** It is a common medical procedure that uses a small dose of radiation to create a picture. The amount of radiation you are exposed to during an X-ray is so small that the risk of any harm to your health is extremely low.

**Electrocardiogram (ECG):** ECG is a safe procedure, and this will be performed periodically to monitor the condition of your heart while you are on this regimen. There is no risk of getting a shock during ECG procedure.

**CT/MRI:** CT (Computed Tomography) is a safe procedure but uses radiation more than the dose of X-ray. This procedure is essential before starting the treatment and during follow up to know the response to treatment. You will be exposed to maximum of two times in the study and hence the risk for your health is low. We may do MRI (Magnetic resonance imaging) if available. This is an extremely safe procedure and has no known health risk.

**6. Benefits to the participant or others**

***Benefits to study participants***

There may or may not be direct benefit to you from participating in this study. All the study medicines, study related tests, and procedures will be provided free of cost to you.

***Benefits to the community***

If we find the study regimen is better than the standard regimen, TBM patients may be treated for a shorter period in the future.

**7. Appropriate alternative therapies available to the participant**

If you are diagnosed with TBM, but do not wish to take part in this study, you will be referred to the National TB Elimination programme (NTEP), a Government Programme for Tuberculosis where you will get treatment for your condition as per the standard practice and current guidelines. If you have questions about alternative treatments, ask the study doctor for additional information.

**8. Precautions while being part of the study**

- All women must avoid getting pregnant while taking the study medications. If you are a woman able to become pregnant (i.e. not sterilized or less than one year since menopause) you should use birth control measures. If a female participant becomes pregnant while on study medications, the risks of continuing the pregnancy will be explained to her. The decision regarding her continuation in the study or withdrawing her from the study will be taken based on her decision to continue the pregnancy. If a woman becomes pregnant during the post treatment follow up period, she will continue in the study. She will also be referred appropriately for obstetric care.
- Breast feeding must also be avoided while on study medications.
- All men should avoid becoming a father to a new child. This is advised as the effects of these medication on the new-born are unknown
- It is not safe to take some medicines while you are having anti-TB medicines. Inform your doctor if you are taking medicines given to you by other health care practitioner

**9. Compensation for study participation**

You will not be given money to take part in this study. However, you will be given financial assistance for the incidental expenses that you may incur during your participation in this study. You will be paid Rs.200/- for travel to the hospital and towards incidental charges during the protocol-specified scheduled visits and Rs.500 on the day of PK visit for the participants who are participating in the PK study. You will not be asked to pay for any tests or study related procedures and they will be done at free of cost.

**10. Compensation for injury while taking part in the study**

In the event of any injury to you while you are participating in this study, you will be provided free medical management as long as required by the Sponsor or till such time it is established that the injury is not related to the clinical study. In the event of study related injury or death, financial compensation by the Sponsor, as decided by the Licensing Authority (Drugs Controller General of India), will be paid to you or to your nominee(s), over and above any expenses incurred on your medical management. You or your nominee(s) have right to contact the Site Investigator/site Ethics Committee for the purpose of making claims in case of study related permanent injury or death.

**11.Confidentially of records**

Your personal information will be kept confidential. You will be identified by an Unique identification number. All the details collected as a part of this study will be kept under lock and key with access only to the study team. We will keep your medical records confidential and only be shared with someone if demanded by an authority as per the law. Your records may be checked by the sponsor and/or its representatives or people from the regulatory authorities and ethics committees to ensure that the study is being carried out correctly. The information from your medical records will not be given to any unauthorized person without your permission. The results of the study might be published in medical journals or presented in conferences. However, your name will not be revealed in any of the publications, presentations or reports which are generated from this study's results.

**12. Withdrawal from study participation and termination**

Participation in this study is purely voluntary. You have the right to withdraw from this study at any time without giving any reasons, without incurring any penalty. If your study treatment is stopped, based on you and your study doctor’s choice, you will be asked to continue to attend the study visits or be otherwise contacted, unless you decide to withdraw from the study completely.

Also, you may be taken off from the study without your consent based on the decision of your doctor. This may happen for the reasons such as, your doctor feels that your continuing participation in the study may be detrimental to your health, or you do not follow the doctor’s instructions. Even if your participation is terminated or the study is terminated there would be no effect on the regular clinical care being offered to you.

**13.Participant’s responsibilities during engagement in the study**

If you agree to participate in the study, you have the following responsibilities.

- You should be willing to sign the informed consent
- You will have to provide your contact details
- You should be willing to provide information as required by the study and keep up study related appointments
- Inform the study staff about any changes in health status or any medications
- You should be cooperative in giving blood samples and attending interview calls as per the schedule
- Not participate in any other research study before the completion of the present study.
- Inform if you prefer to withdraw from the study

**14. Availability of new information and participant's willingness to continue in the study**

It is possible that during the course of this study, new information becomes available about the drugs that are being studied. If this happens, your doctor will tell you about it and discuss with you whether you want to continue in the study, if you decide to withdraw your doctor will make arrangements for your care to continue. If you decide to continue in the study you will be asked to sign an updated consent from. Also, on receiving new information your research doctor might consider it to be in your best interests to withdraw or change your treatment regimen in the study. He/she will explain the reasons and arrange for your care to continue.

**15. Storage of samples:**

The blood and CSF samples collected for the study shall be analyzed and the leftover samples may be stored for future use in research after obtaining your consent. In addition, samples collected for future research will also be stored with unique numbers without personal identifiers for a maximum period of 10 years.

**16. Contact for study related queries, rights of participant and in the event of any injury**

If you have any questions or concerns related to the study, you can contact the Site Principal Investigator. For any issues related to your rights as research participant you can contact Member Secretary, NIRT- Ethics Committee at 044-28369567 during working hours (9 am -5 pm, Monday to Friday).The names and contact details are provided below for the NIRT investigators.

|  | **Name** | **Phone** | **Email** |
| --- | --- | --- | --- |
| NIRT investigators | Dr Leeberk Raja | 9611770181 | leeberk.raja@icmr.gov.in |
|  | Dr Bella Devaleenal | 9841746690 | belladevaleenal.d@icmr.gov.in |

**Informed consent form**

**Comparative evaluation of intensified short course regimen and standard regimen for adults TB meningitis : an open label randomized controlled trial (INSHORT trial)**

I ______________________have read / been read out the patient information sheet of the study titled**: Comparative evaluation of intensified short course regimen and standard regimen for adults TB meningitis : an open label randomized controlled trial (INSHORT trial)**

I have discussed with the study investigators about the purpose of the study, the procedures involved, the expected risks and benefits involved, participant safety procedures and protection of rights of study participants in detail.

I have been given the opportunity to ask questions, which have been answered to my satisfaction. I understand that any questions that I might have will be answered verbally or if I prefer, with a written statement.

I understand that my participation in this study is voluntary and that I may refuse to participate. I also understand that if, for any reason, I wish to discontinue my participation in this study at any time, I will be free to do so without my medical care or legal rights being affected.

I understand that the sponsor of the study or its representative, ethics committee and regulatory authorities will access my health records both in respect of the current study and further research that may be conducted in relation to it, even if I withdraw from the study. Also my identity, medical records, and data relating to this research study will be kept confidential, except as required by law. If I have any questions concerning my rights as a research participant in this study, I may contact any of the study investigators at any time point.

I agree not to restrict the use of any data or results that arises from this study provided such as a use for scientific purpose(s).As I am fully informed of the study, its risks and benefits, I hereby consent to participate in the procedures set forth. Also, I have received a copy of the Patient information sheet and consent form.

**Use of specimen for future research**

| I consent to have my blood/plasma/serum/ sputum/ CSF sample and data stored to be used for future research studies, without consulting me again |  |  |
| --- | --- | --- |
| I consent to have my blood/plasma/serum/sputum /CSF sample and data stored to be used for future research studies, after consulting me again |  | |
| I do not consent for my blood/plasma/serum/sputum /CSF sample and data to be stored to be used for future research studies |  |  |

**Declaration**

I _____________________________ have read / understood the consent form and I understand all the information given. I have also understood the terms and conditions of the study and my rights about the study. I wish to participate in this study of my own free will and give my consent to participate in this study.

_______________________ ____________________________ ____. ____

Participant’s Name Participant’s Signature / Thumb impression Date

___________________ _____________________________ ____ ______

Witness’s Name (if needed) Witness’s signature Date

___________________________________________________________________________

Legally authorized or acceptable (LAR) relative name Signature of LAR Date

(if the participant is very sick)

___________________________________________________________________________

Name of person obtaining consent Signature of the person obtaining consent Date

___________________________________________________________________________

Site Investigator Site Investigator / Date

Representative Name Representative Signature
